# Checkpoint Inhibition Reduces the Threshold for Drug-Specific T-Cell Priming and Increases the Incidence of Sulfasalazine Hypersensitivity

**DOI:** 10.1093/toxsci/kfab144

**Published:** 2021-11-29

**Authors:** Sean Hammond, Anna Olsson-Brown, Sophie Grice, Andrew Gibson, Joshua Gardner, Jose Luis Castrejón-Flores, Carol Jolly, Benjamin Alexis Fisher, Neil Steven, Catherine Betts, Munir Pirmohamed, Xiaoli Meng, Dean John Naisbitt

**Affiliations:** 1 D epartment of Molecular and Clinical Pharmacology, MRC Centre for Drug Safety Science, University of Liverpool, Liverpool L69 3GE, UK; 2 ApconiX, Alderley Edge SK10 4TG, UK; 3 Institute for Immunology and Infectious Diseases, Murdoch University, Murdoch, Western Australia, WA 6150, Australia; 4 Instituto Politécnico Nacional, Unidad Profesional Interdisciplinaria de Biotecnología, Mexico City 07340, México; 5 Institute of Inflammation and Ageing, College of Medical and Dental Sciences, University of Birmingham, Birmingham, UK; 6 National Institute of Health Research (NIHR) Birmingham Biomedical Research Centre, Birmingham, UK; 7 Department of Rheumatology, University Hospitals Birmingham NHS Foundation Trust, Birmingham, UK; 8 Institute of Immunology and Immunotherapy, College of Medical and Dental Sciences, University of Birmingham, Birmingham, UK; 9 Cancer Centre, University Hospitals Birmingham NHS Foundation Trust, Birmingham, UK; 10 Pathology Sciences, Drug Safety and Metabolism, IMED Biotech Unit, AstraZeneca, Cambridge, UK

**Keywords:** immune checkpoint inhibitor, drug hypersensitivity, T lymphocytes, sulfasalazine, immune-related adverse events

## Abstract

An emerging clinical issue associated with immune-oncology agents is the collateral effects on the tolerability of concomitant medications. One report of this phenomenon was the increased incidence of hypersensitivity reactions observed in patients receiving concurrent immune checkpoint inhibitors (ICIs) and sulfasalazine (SLZ). Thus, the aim of this study was to characterize the T cells involved in the pathogenesis of such reactions, and recapitulate the effects of inhibitory checkpoint blockade on de-novo priming responses to compounds within in vitro platforms. A regulatory competent human dendritic cell/T-cell coculture assay was used to model the effects of ICIs on de novo nitroso sulfamethoxazole- and sulfapyridine (SP) (the sulfonamide component of SLZ) hydroxylamine-specific priming responses. The role of T cells in the pathogenesis of the observed reactions was explored in 3 patients through phenotypic characterization of SP/sulfapyridine hydroxylamine (SPHA)-responsive T-cell clones (TCC), and assessment of cross-reactivity and pathways of T-cell activation. Augmentation of the frequency of responding drug-specific T cells and intensity of the T-cell response was observed with PD-1/PD-L1 blockade. Monoclonal populations of SP- and SPHA-responsive T cells were isolated from all 3 patients. A core secretory effector molecule profile (IFN-γ, IL-13, granzyme B, and perforin) was identified for SP and SPHA-responsive TCC, which proceeded through Pi and hapten mechanisms, respectively. Data presented herein provides evidence that drug-responsive T cells are effectors of hypersensitivity reactions observed in oncology patients administered ICIs and SLZ. Perturbation of drug-specific T-cell priming is a plausible explanation for clinical observations of how an increased incidence of these adverse events is occurring.

The emergence of immune checkpoint inhibitors (ICIs) as a therapeutic option has dramatically altered the landscape of oncological treatment. The outstanding efficacy observed with the use of an assortment of antibodies targeted at the PD1 and CTLA-4 co-inhibitory pathways (Hargadon *et al.*, 2018) has demonstrated the potential of this approach even within its nascent stages. Unfortunately, advancements in immuno-oncology have been accompanied by the advent and rise of novel adverse drug reactions, coined immune-related adverse events (irAEs). These irAEs encompass a plethora of events with a heterogeneous spectrum of presentations including dermatologic, GI, endocrine, hepatic, respiratory, renal, hematological, ocular and CNS manifestations, many of which are thought to be attributable to the immune dysregulation imposed by the action of ICIs favoring autoimmune or otherwise pathological deployment of T cells (Michot *et al.*, 2016).

This altered immunological perception of self and tumor antigens immediately brings into question whether ICI therapy also perturbs the body’s defences against concomitantly administered xenobiotics. Indeed, several studies have shown associations between microbiome composition and the efficacy/toxicity profiles of ICIs (Chaput *et al.*, 2017; Dubin *et al.*, 2016; Gori *et al.*, 2019; Pezo *et al.*, 2019), which may reflect discrepancies in the tolerance thresholds to particular microbial organisms. Moreover, this shift in immunological perception also holds true with organ transplant recipients as ICIs are poorly tolerated in such individuals, eliciting organ rejection in a high proportion of individuals (Kittai *et al.*, 2017). An extension of, and important facet of this ICI-mediated dysregulation is the collateral effects on the immune-toxicological profiles of concomitant medications. This concept has been adeptly illustrated by the generation of a mouse model which reportedly exhibits an idiosyncratic drug-induced liver injury (IDILI)-like phenotype upon exposure to IDILI causing drugs, such as amodiaquine, isoniazid, and nevirapine (Mak and Uetrecht, 2015; Metushi *et al.*, 2015).

In clinical settings, hypersensitivity rates to compounds have been exacerbated by the concomitant administration of ICIs (Cui *et al.*, 2020; Ford *et al.*, 2018; Kimura *et al.*, 2020; Koda *et al.*, 2018; Shirali *et al.*, 2016; Uhara *et al.*, 2018; Yamazaki *et al.*, 2015). A prominent example of this is sulfasalazine (SLZ), which is metabolized by colonic bacteria to its constituents, the sulfonamide antibiotic sulfapyridine (SP) and the anti-inflammatory mesalazine (5-ASA). Ironically, SLZ’s anti-inflammatory properties have prompted its use in amelioration of irAEs (Chan *et al.*, 2015; Lomax *et al.*, 2018; Naidoo *et al.*, 2017). However, in one of the first reports to highlight an immunologically driven drug-drug interaction between ICIs and concomitant medication, SLZ was flagged as poorly tolerated in individuals receiving anti-PD-1 checkpoint blockade (Ford *et al.*, 2018). Herein, we recapitulate the effects of inhibitory checkpoint blockade on de novo priming responses to sulfonamides within in vitro platforms utilizing peripheral blood mononuclear cells (PBMC) from healthy blood donors and present functional and phenotypic characterization of effector T cells involved in the pathogenesis of SLZ hypersensitivity reactions in patients on ICIs.

## MATERIALS AND METHODS

###  

####  

##### Procurement of reagents and chemicals

Nitroso sulfamethoxazole (synthesized as described in Naisbitt *et al.* [1996]) was purchased from Synthesis MED CHEM Ltd (Cambridgeshire, UK). Sulfapyridine hydroxylamine (SPHA) was synthesized in accordance with methods described in Castrejon *et al.* (2010b). Nitroso SP is unstable and difficult to synthesize in a pure form. However, SPHA auto-oxidizes in aqueous solution to the protein-reactive nitroso metabolite. Hence, it is important to note that T cells are exposed to nitroso SP in cultures labeled SPHA. SLZ, SP, and 5-ASA were purchased from Sigma-Aldrich (Gillingham, UK).

##### Human subjects

Blood was collected from sulfonamide-naïve healthy donors with approval from the Liverpool Research Ethics Committee to study de novo T-cell priming responses. All participants gave written informed consent before the research commenced. Three SLZ -treated patients were also recruited as cases to the A Mechanistic Investigation into Drug and Chemical Induced Hypersensitivity Reactions (HYST) study (12/NW/0525) following initial identification and diagnosis as described in Ford *et al.* (2018) (Table  1). The patients gave written informed consent. Up to 120 ml of venous blood was collected from each participant for isolation of PBMC via gradient-density separation. Tolerant patients were not included in the study as SLZ is now contraindicated in this patient group,

**Table 1.** Clinical Characteristics of Sulfasalazine Hypersensitive Patients

	Gender	Age	Immunotherapy Indication	Previous Immunotherapy	Immunotherapy at Time of Reaction	Indication for Sulfasalazine	Duration on Sulfasalazine (days)	Clinical Presentation of Sulfasalazine Reaction	Ameliorative Measures	LTT Result	Drug Specific T Cells
Patient 1	Male	57	Metastatic melanoma	Ipilimumab. (stopped due to hypophysitis and disease progression)	Pembrolizumab	Monoarthritis (subsequently symmetrical oligoarthritis of knees and ankles)	10	Fever, erythematous maculopapular rash across face, neck, chest, and back, acute rise in ALT and CRP and lymphopenia	Discontinuation of pembrolizumab and sulfasalazine, IV methylprednisolone (3 days) followed by oral prednisolone wean	SPHA (SI 2.8, 40 µM)	SPHA, possible SP
Patient 2	Male	51	Metastatic melanoma	Ipilimumab. (stopped due to hypophysitis and hepatitis)	Pembrolizumab	Monoarthritis (subsequently symmetrical oligoarthritis of knees and ankles)	(A) 5, (B) 11, (C) 9, (D) 21—concomitant steroid	(A) Fever, cough, raised CRP. (B) Fever, raised CRP. (C) Fever, nonproductive cough, raised CRP. (D) nausea, diarrhea, raised ALT and CRP	Discontinuation/deferral of pembrolizumab and discontinuation of sulfasalazine, oral prednisolone	SP (SI 15.2, 200 µM) and SPHA (SI 3.2, 50 µM)	SPHA and SP
Patient 3	Male	77	Metastatic melanoma	Ipilimumab. (stopped due to progression)	Pembrolizumab	Symmetrical polyarthritis	35	Acute late onset colitis	Discontinuation of pembrolizumab and sulfasalazine, high dose steroids	Possible SLZ (SI 2.1, 200 µM)	SPHA and SP

Abbreviations: CRP, C-reactive protein; ALT, alanine aminotransferase; SLZ, sulfasalazine; SP, sulfapyridine; SPHA, sulfapyridine hydroxylamine; LTT, lymphocyte transformation test; SI, stimulation index.

##### De novo drug-specific T-cell priming with PBMC from healthy donors

T-cell multiwell priming assays were conducted as described in Ogese *et al.* (2020) with modification to generate a more regulatory competent model. Specifically, whole purified T-cell populations were utilized at the coculture stage, rather than isolated naïve T cells, thus incorporating T-regulatory cells, known to be important for peripheral tolerance mechanisms. These adapted priming assays entailed a 12-day coculture of monocyte-derived dendritic cells (8 × 10^3^/well) with whole T cells (1 × 10^5^/well) in the presence of the model sulfonamide hapten nitroso sulfamethoxazole (20–40 µM) or SPHA (20–40 µM) (up to 96 cultures were established in 96-well plates, depending on availability of cells). Recall responses were then assessed through rechallenge with media control or relevant drug metabolite for 48 h, with proliferative responses measured by virtue of incorporation of [^3^H] thymidine (0.5 µCi/well, 5 Ci/mmol; Moravek Biochemicals, Brea, California) during an additional 16 h. For investigation into the effects of immune-checkpoint blockade within priming cocultures, commercially available antibodies (anti-PDL-1; 5 µg/ml IgG2b mAb, clone 29E.2A3, anti-PD-1; 5 µg/ml IgG1 mAb, clone EH12.2H7, both Biolegend UK) were incorporated into assays >1 h prior to the addition of drug at the priming stage. We present data as raw cpm values in each individual well (24 per culture condition) alongside pie charts where the response of individual wells is categorized according to the strength of response (stimulation index [SI] less than 1.5, 1.5–2.5, 2.5–4, 4–10, and greater than 10). Statistical analysis of the dataset was not performed as you will never stimulate all wells in this form of experiment. This is because some cultures do not contain precursor cells that can be activated with the drug. Hence, a number of nonresponding wells is observed alongside responding wells showing varying degrees of response.

##### Initial assessment of hypersensitive patient lymphocyte responses to sulfasalazine and its metabolites using the lymphocyte transformation test

Hypersensitive patient PBMCs (1.5 × 10^5^/well) were cultured for 5 days (37°C 5% CO_2_) with various concentrations of SLZ (10–400 µM), 5-ASA (10–400 µM), SP (10–400 µM), SPHA (2.5–50 µM) or phytohemagglutinin (PHA; positive control). Cultures were then pulsed with [^3^H]-thymidine (0.5 µCi/well) incubated for a further 16 h after which antigen-induced proliferative responses were measured via ensuing thymidine incorporation using established methods (Pichler and Tilch, 2004).

##### Isolation of compound-responsive T-cell clones

Bulk, compound response T-cell-enriched cultures were generated via 14 day culture of patient PBMC (2 × 10^6^ cells/well; 1 ml) in the presence of SP (50–200 µM) or SPHA (20–50 µM), in R9 medium (RPMI 1640 supplemented with 10% human AB serum [Class A; Innovative Research Inc., Novi, Michigan], 25 mM of HEPES, 10 mM of l-glutamine, and 25 mg/ml of transferrin [Sigma-Aldrich]). Cultures were supplemented with 200 IU/ml of recombinant human interleukin (IL-2) (PeproTech, London, UK) on days 6 and 9. Monoclonal populations of T cells (T-cell clones, TCC) were isolated from the resulting enriched cell lines via limiting dilution, as described previously (Sullivan *et al.*, 2018). PBMC from patients were cultured in supernatant from an Epstein-Barr virus (EBV)-producing cell line (B95.8), in order to generate EBV-transformed B-cell lines. The resulting EBVs then served as immortalized source of autologous antigen presenting cells (APC), which were maintained in “F1” media (RPMI1640 supplemented with 10% fetal bovine serum [Invitrogen, Paisley, UK], 100 mM l-glutamine, penicillin, and streptomycin).

Following limiting dilution, TCC were subject to repetitive mitogen-driven expansion (PHA 5 mg/ml, IL-2 200 IU/ml, irradiated, allogeneic PBMCs 1 × 10^4^ cells/well). Upon satisfactory expansion, specificity screening of TCC was conducted using a coculture format of 5 × 10^4^ TCCs and 1 × 10^4^ autologous irradiated EBV-transformed B-cells per well (96-well U bottomed) incubated with relevant compound for 48 h. Cultures were then pulsed with [^3^H] thymidine (0.5 µCi/well, 5 Ci/mmol; Morovek Biochemicals, Brea, California) for a further 16 h, with radioactivity incorporation during this period serving to measure compound specific TCC proliferation. TCC exhibiting stimulation indices (SIs) (proliferation in the presence of compound/proliferation in control wells) surpassing 1.5 at this stage were deemed compound-responsive and were subject to an additional round of mitogen-driven expansion in order to facilitate further characterization.

##### Dose response and cross-reactivity of T-cell clones

The dose dependency and compound specificity of proliferation responses for SP and SPHA TCC were evaluated using cocultures (5 × 10^4^ TCC, 1 × 10^4^ irradiated autologous EBV-transformed B-cells) exposed to SP (10 nM–1.5 mM) and SPHA (1 nM–50 μM) with alternate compounds used at a minimum of 2 stimulatory concentrations. Cross-reactivity of TCC was also investigated for sulfamethoxazole (100 nM–200 µM) and its nitroso metabolite (1–50 μM).

##### Cellular phenotyping

TCC were phenotyped as CD4/8 using respective fluorochrome-conjugated antibodies (BD Biosciences) with flow cytometry conducted on a FACSCanto II instrument (BD Biosciences).

##### Profiling of secretory molecule release upon antigen challenge

Enzyme-linked immunospotting (ELISpot) methods were employed to evaluate the profile of secretory molecules (IFN-γ, IL-13, IL-17, IL-22, granzyme B, and perforin) released by TCC upon antigenic challenge. Plates were precoated with appropriate capture antibody for 24 h, cocultures (5 × 10^4^ TCC, 1 × 10^4^ irradiated autologous EBV-transformed B cells) were incubated in the presence/absence of SP or SPHA at various concentrations for 48 h, after which plates were washed and secreted molecules were visualized using an AID ELIspot reader (Oxford Biosystems Cadama, Oxfordshire, UK) in line with manufacturer’s instructions (Mabtec).

##### Mechanistic studies of antigen presentation

The dependency of T-cell activation on HLA molecules was evaluated through blocking antibodies directed at class I (clone W6/32, 5 µg/ml) or II (HLA-DR; B7/21, HLA-DP; LB3.1, HLA-DQ; SVP-L3 5 µg/ml each) included in cocultures (5 × 10^4^ TCC, 1 × 10^4^ irradiated autologous EBV-transformed B-cells) treated with optimal stimulatory concentrations of drug. Pretreatment of autologous EBV-transformed B cells prior to inclusion in standard coculture assays was used to determine the pathways of drug presentation as described in Hammond *et al.* (2020). Glutaraldehyde fixation (0.05%; Sigma Aldrich) was used to terminate metabolic processes/intracellular processing, whereas prepulsation (30 min–24 h) with optimal concentrations of SP or SPHA, followed by extensive washing was used for evaluation of the dependence on soluble drug. All cultures were subject to 48 h incubation, followed by pulsation with [^3^H] thymidine (0.5 µCi/well, 5 Ci/mmol) and a further 16 h incubation period. Incorporation of radioactivity was used to measure proliferative responses.

##### Glutathione trapping of hydroxylamine metabolite

Glutathione trapping experiments were conducted with direct addition of SPHA to glutathione in aqueous solution (100 nM–10 mM glutathione and SPHA, 24 h, 37°C). The resulting products were diluted with 0.1% formic acid (1:10 dilution) and samples were delivered into a 5500 QTRAP (AB Sciex, Framingham, Massachusetts) via infusion.

## RESULTS

###  

#### Enhanced Drug-Specific De Novo T-Cell Priming Responses Are Seen Upon Addition of Checkpoint Blocking Antibodies

**Figure 1.**
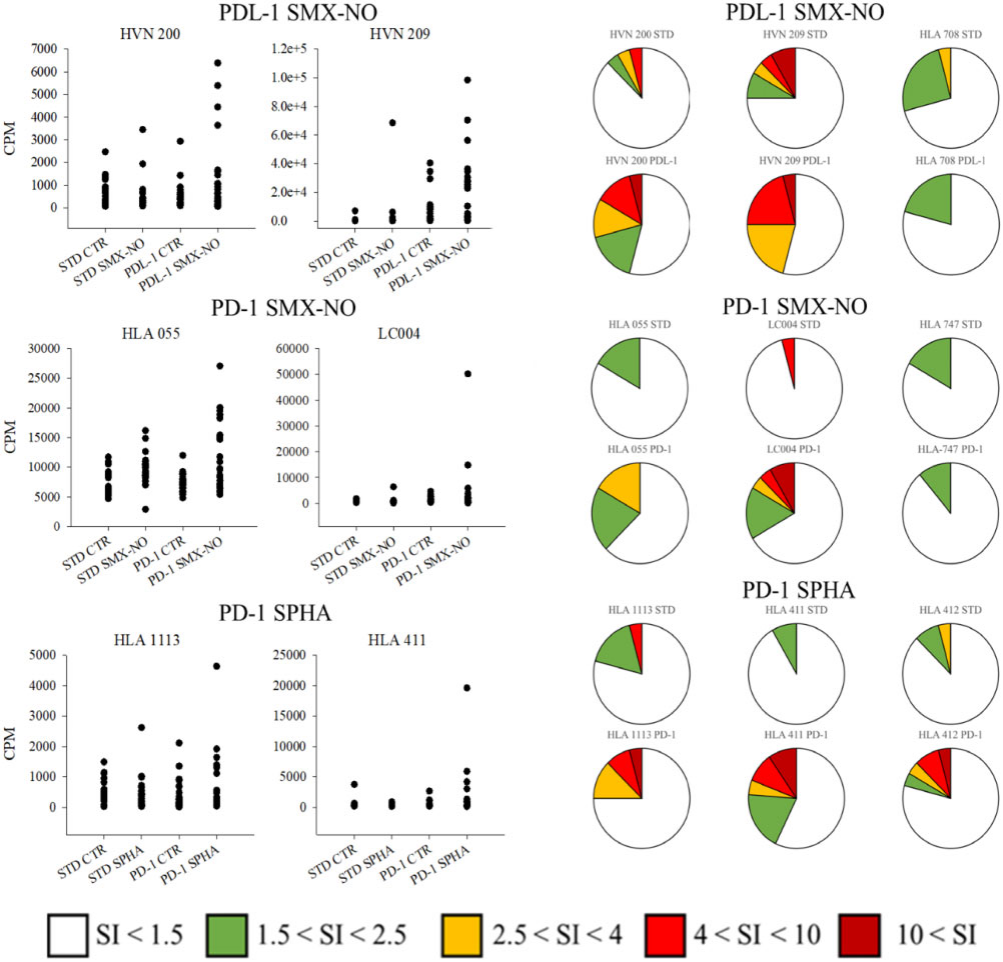
Assessment of ICI perturbation of de novo T-cell priming responses to the model antigen sulfamethoxazole nitroso (SMX-NO) and the hydroxylamine metabolite of sulfapyridine (SP). (Top) Representative priming responses to SMX-NO in standard and PDL-1 blockade cultures. (Middle) Relative priming responses to SMX-NO in standard and PD-1 blockade cultures. (Lower) Priming responses to SPHA in standard and PD-1 blockade cultures. Priming cocultures (whole T-cell populations 2 × 10^5^, autologous monocyte derived DC 8 × 10^3^) were exposed to SMX-NO (20–40 µM) or SPHA (20–40 µM) in the presence or absence of PD-1 or PDL-1-directed blockade antibodies (5 µg/ml). After 12 days of incubation (37°C, 5% CO_2_), plates were washed repeatedly to remove soluble antigens. Cultures were then rechallenged with media control or the relevant antigen for 48 h, then pulsed with [3H]-thymidine (0.5 µCi/well) for a further 16 h. Incorporation of radioactivity then served as a measure of proliferation within cultures. All conditions were performed with 24 replicates. Left-hand side: data presented as dot plots. Right-hand side: data presented as pie charts depicting breakdown of responses for drug-treated cultures as a function of stimulation indices (SIs) of the mean value for 24 replicates of media control cultures. Response classification within pie charts; negative response (SI < 1.5), weak response (1.5 < SI < 2.5), moderate response (2.5 < SI < 4), strong response (4 < SI < 10), and extreme (10 < SI).

Proliferative responses following priming and rechallenge of naïve T cells from healthy donors with nitroso sulfamethoxazole were enhanced with in vitro administration of PDL-1 (4/5 donors) (Figure  1, 2 positive and 1 negative response shown). As highlighted above, this modified version of the T-cell priming assay utilized whole T-cell populations containing Tregs to more closely replicate physiological conditions; hence, only weak responses were detected with nitroso sulfamethoxazole in the absence of immune checkpoint inhibition. PD-1 blockade enhanced responses in 3/5 donors. ICI blockade of cultures yielded greater percentages of responding wells, generally of greater intensity; as an example, HLA-647 exhibited responses in 25% (STD), 42% (PDL-1) and 29% (PD-1). Application of PD-1 blockade priming assays to model the patient scenario yielded enhanced responses in 4/5 individuals for SPHA (Figure  1).

**Figure 2.**
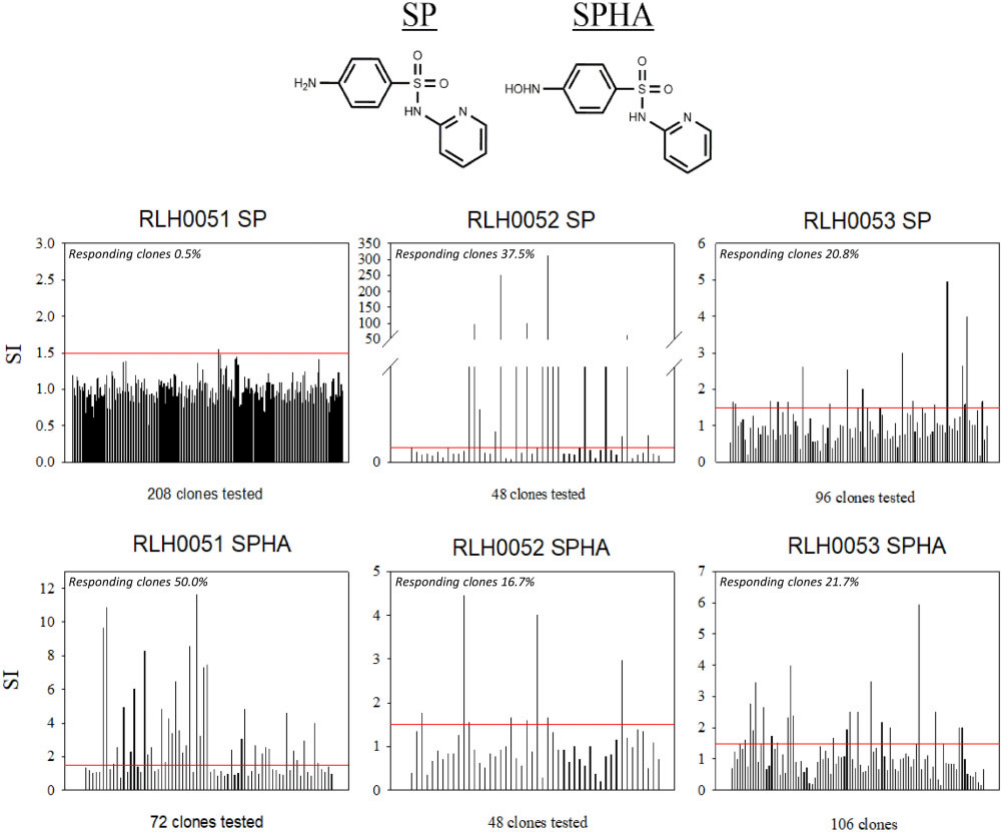
Identification of SP and sulfapyridine-hydroxylamine (SPHA) antigen-responsive T-cell clones (TCC) from sulfasalazine (SLZ) hypersensitive patients. (Top) Chemical structures of SLZ, mesalazine (5-ASA), SP, and SPHA. (Bottom) TCC were isolated via limiting dilution of bulk enrichment cultures and subjected to repetitive mitogen stimulation for expansion. Compound responsivity screening entailed coculture of TCC (0.5 × 10^5^) and autologous EBV-transformed B cells (0.1 × 10^5^) in the presence and absence of compounds (SP or SPHA) for 48 h (37°C, 5% CO_2_). [^3^H]-thymidine (0.5 µCi/well) was added for the last 16 h of incubation before plates were harvested and proliferation measured. Individual bars represent mean drug-treated/mean media control proliferation responses. TCC exhibiting a proliferation stimulation index (SI) of 1.5 or above (as denoted by red lines) which were deemed specific at this stage and were expanded for further investigation.

#### Identification of Drug-Specific Lymphocyte Responses Within Patient PBMC

Three patients that developed hypersensitivity when exposed to pembrolizumab and SLZ were recruited to the study. The clinical cases are discussed in detail in Ford *et al.* (2018) and summarized in Table  1. SLZ was discontinued on clinical presentation of the adverse event and steroids were used as ameliorative measures. Patient PBMCs were stimulated to proliferate in the presence of SP (patient 2) and/or its hydroxylamine metabolite (patients 1 and 2) as determined by positive lymphocyte transformation test responses (SI >2.5). Weakly positive patient 3 PBMC proliferative responses were detected for SLZ (SI 2.1) albeit with the caveat of low baseline CPM counts. To confirm the PBMC data, T-cell enrichment and subsequent limiting dilution was performed for SP and SPHA. Approximately 5400 cultures were generated from all 3 patients, giving rise to 352 (SP) and 226 (SPHA) TCC for specificity screening, which in turn yielded CD4+ SP and SPHA-responsive TCC in all 3 patients (Figure  2).

#### Dose-Response and Cross-Reactivity Profiles of TCC

**Figure 3.**
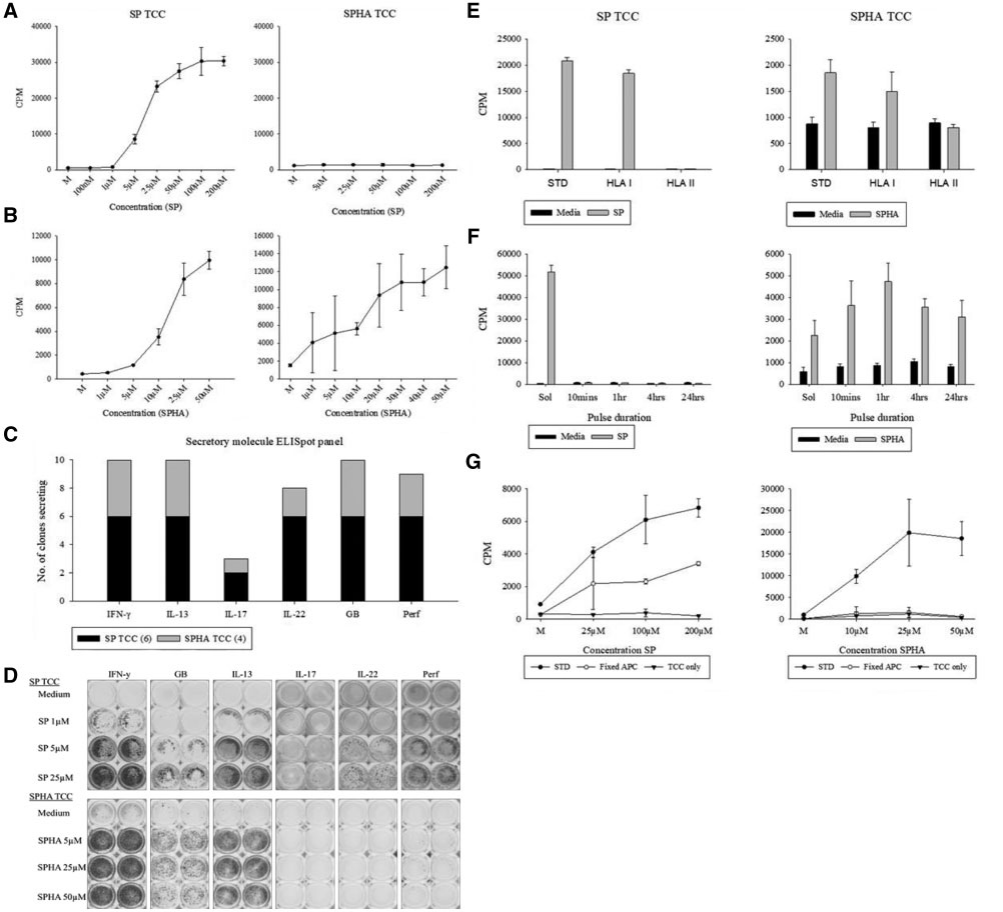
Characterization of SP and SPHA-responsive TCC derived from SLZ hypersensitive patients. A, B, Dose-response curves and cross-reactivity series for representative SP (left-hand side) and SPHA (right hand side)-responsive TCC to SP (A) and SPHA (B). C, Overview of secretory molecule profiles of 10 compound-responsive TCC upon stimulation with SP (4) or SPHA (6). D, ELISpot well images depicting dose-dependent secretory patterns of representative TCC responsive to SP and SPHA relative to medium-treated control cultures. TCC (5 × 10^4^) were cultured with autologous EBV-transformed B-cells (1 × 10^4^) and titrated concentrations of SP or SPHA for 48 h at 37°C, 5% CO_2_. For proliferative studies (A and B) [3H]-thymidine (0.5 μCi/well) was added for the last 16 h of incubation before plates were harvested and proliferation assessed via ensuing incorporation. Data presented as mean CPM ± standard deviation. Cytokine secretion (C and D) was measured by ELISpot. E, Cocultures (5 × 10^4^ TCC, 1 × 10^4^ irradiated autologous EBV-transformed B cells) were exposed to SP or its SPHA in the presence or absence of an MHC blocking antibodies. SP and SPHA responses were reduced to medium control values with HLA class II block indicating the SP and SPHA-specific T-cell activation is HLA class II restricted. F, EBV-transformed B cells (1 × 10^4^) were pulsed with SP or SPHA for 10 min–24 h, then washed repeatedly to remove unbound drug prior to coculture with TCC (5 × 10^4^). G, TCC (5 × 10^4^) were cultured with SP/SPHA alone (TCC only), in the presence of glutaraldehyde-fixed (1 × 10^4^) (fixed APC), or irradiated EBV-transformed B cells (1 × 10^4^) (STD). All cultures were subject to a 48-h incubation period 37°C, 5% CO_2_, after which wells were pulsed with [3H]-thymidine (0.5 μCi/well) for an additional 16 h incubation. Plates were then harvested and proliferation assessed through incorporated radioactivity. Data presented as mean CPM ± standard deviation.

TCC exhibited a dose-dependent proliferative response to SP (1–200 µM) and SPHA (1–50 µM) up to the respective toxicity thresholds. Unidirectional cross reactivity was observed with SP-responsive TCC exhibiting additional reactivity to SPHA whilst the inverse was not observed; representative TCC are depicted in Figs.  3A and 3B). No additional cross-reactivity was observed with exposure to the structurally related sulfonamide antibiotic sulfamethoxazole, its metabolites sulfamethoxazole hydroxylamine, and nitroso sulfamethoxazole or 5-ASA (data not shown).

#### Profile of Secretory Molecules Released Upon TCC Activation

A panel of 10 TCC (6 SP, 4 SPHA) were subject to ELISpot in order to identify cytokines and cytolytic molecules released upon antigenic challenge. A core secretory pattern of IFNγ, IL-13, and granzyme B were identified across all TCC. Perforin and IL-22 were detected from all SP-responsive TCC and 3/4 SPHA TCC, whilst IL-17 secretion was identified in a minority of both SP- and SPHA-responsive TCC (Figure  3C). As with the proliferative responses, cytokine secretion occurred in a dose-dependent fashion as illustrated by the representative TCC in Figure  3D.

#### Sulfapyridine and Sulfapyridine Hydroxylamine-Associated Antigens Are Recognized by TCC in the Context of HLA and Proceed Through Pi and Hapten Mechanisms Respectively

Abrogation of T cells responses by HLA blocking antibodies demonstrated that compound-associated antigens were generally presented in an HLA class II restricted fashion (Figure  3E). APC pulsing and fixation experiments have been used previously to differentiate between the 3 mechanisms of antigen presentation (hapten, Pi, and altered self-repertoire) as depicted by distinct profiles of model compounds in Hammond *et al.* (2020). In parallel to sulfamethoxazole and its hydroxylamine and nitroso metabolites (Naisbitt *et al.*, 1996), distinct profiles were observed for TCC responsive to SP and SPHA. Responses for SP-responsive TCC were dependent on the continuous presence of soluble drug as demonstrated by the absence of proliferation for all pulse durations, whilst reintroduction of soluble drug reinstated activation. By contrast, SPHA-responsive TCC were activated by all pulsation time points, indicating a covalent mechanism for T-cell activation (Figure  3F). Glutaraldehyde fixation of APC prior to coulture is an established technique for evaluating the role of intracellular antigen processing and metabolic processes in antigen presentation. Prefixation of APC prior to coculture abrogated responses of SPHA-responsive TCC, whilst considerable activation was retained with SP TCC (Figure  3G). Fixation/pulsing experiments were also performed to investigate the mechanism by which SP TCC were cross-reactive for SPHA, with the same profile observed as for SP itself (negative pulsing, mostly impervious to the effects of fixation, data not shown).

#### Sulfapyridine Hydroxylamine Forms Adducts With Glutathione

**Figure 4.**
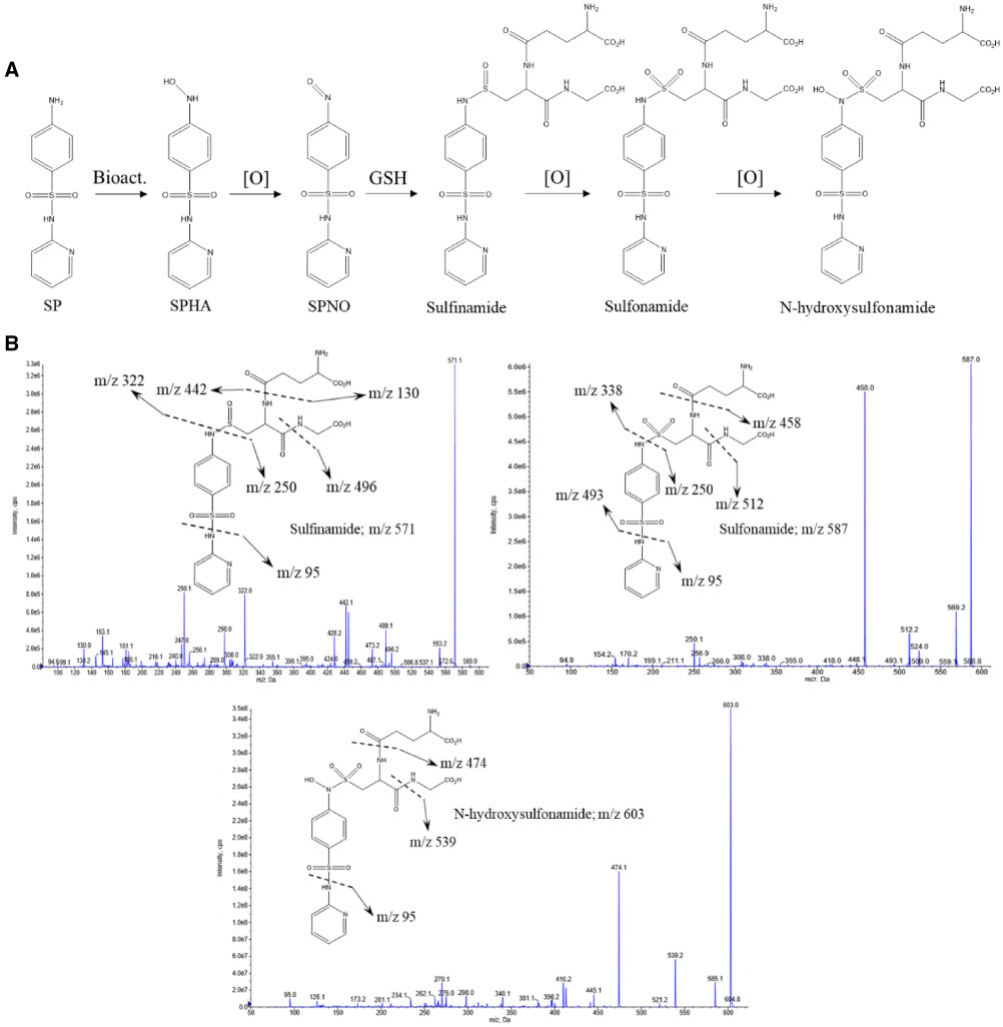
Mass spectrometric characterization of SPHA/nitroso adducts. A, Putative scheme of derivation of reactive hydroxylamine and nitroso metabolites from parent drug SP and all possible downstream adducts with glutathione. B, Mass spectrometric confirmation of glutathione adducts, MS/MS spectra depict the formation of sulfinamide (*m/z* 571), *N*-hydroxysulfinamide and sulfonamide (*m/z* 587), and *N*-hydroxysulfonamide (*m/z* 603) adducts.

Evidence for thiol reactivity of SPHA and thus similar adduction chemistry to that observed with metabolites derived from other aromatic amine containing sulfonamides such as nitroso sulfamethoxazole was provided through the detection of parallel adducts, namely sulfinamide (*m*/*z* 571), *N*-hydroxysulfinamide and sulfonamide (*m*/*z* 587), and N-hydroxysulfonamide (*m*/*z* 603) adducts (Figure  4).

## DISCUSSION

Oncology patients treated with ICIs represent a cohort of individuals in whom polypharmacy is common (Gandhi *et al.*, 2020; McGahey and Weiss, 2017). An important question to address is whether the introduction of immune deregulating agents can heighten the immunological perception of drugs, and whether this translates to greater hypersensitivity rates. The reporting of augmented hypersensitivity rates in patients given the codrug SLZ (Ford *et al.*, 2018) was of particular interest as it has relatively well-known liabilities for hypersensitivity, eliciting a broad range of manifestations including the spectrum of cutaneous reactions (Leroux *et al.*, 1992; Raithatha *et al.*, 2014), DRESS (Aquino *et al.*, 2008), and hepatotoxicity (Losek and Werlin, 1981; Ribe *et al.*, 1986; Sotolongo *et al.*, 1978). Both of the SLZ constituents have been implicated in adverse reactions and as a result, caution is required with both aminosalicylates/acetylsalicylic acid and sulfonamides once SLZ hypersensitivity has been identified due to concerns over potential cross reactivity. Whilst there are instances where 5-ASA is the causative agent (Hautekeete *et al.*, 1992; Ransford and Langman, 2002), the majority of SLZ reactions are thought to arise due to the SP component (Giaffer *et al.*, 1992; Rao *et al.*, 1987; Turunen *et al.*, 1987). Indeed, reports illustrating SP’s culpability in such reactions span back to its market authorization as a stand-alone pharmaceutical (Davidson and Bullowa, 1940). As for several other sulfonamides, considerable evidence has been garnered for the involvement of T cells in these reactions, with positive clinical diagnostics and lymphocyte stimulation assays (Zawodniak *et al.*, 2010; Castrejon *et al.*, 2010a).

We therefore focused initially on whether ICI affect de novo drug-specific T-cell responses. Previous studies incorporating ICIs into in vitro priming assays have yielded priming responses of greater intensity (Ogese *et al.*, 2020), though readouts have been accompanied and undermined by baseline drift. Thus, a logical deduction in the apparent predisposition of ICI patients to hypersensitivity is that throughout the duration of ICI therapy the blockade of immune checkpoints would temporally deregulate priming responses to drugs. To explore this, we utilized a more immune-regulatory competent iteration of the T-cell priming assay. The assay includes T-regulatory cells which are known to be important in peripheral tolerance mechanisms (Gibson *et al.*, 2017a), and have been shown to regulate in vitro drug-specific T-cell priming (Gibson et al., 2017b) Features of the revised T-cell priming assay included lower baseline proliferation and the failure of around one quarter of assays conducted with the model compound nitroso sulfamethoxazole and SPHA to exhibit convincing priming responses, which is presumably due to the action of T-regulatory cells. An enhanced manifestation of priming responses was observed with nitroso sulfamethoxazole when PD-directed blockade was added. We then proceeded to model the clinical scenario that occurred within SLZ hypersensitive patients in an in vitro T-cell priming setting, utilizing PD-1 blockade and SPHA. Enhanced priming across most donors was observed with addition of the PD-1 blocking antibody, indicating that T-cell responses to associated drugs is altered unfavorably.

Whilst the enhanced de novo priming of SP-specific T cells provides mechanistic insight into how ICI administration may contribute to the outcomes observed in patients, the data does not confirm that the adverse events have an immune pathogenesis Hence, we utilized PBMC from the patients reported by Ford *et al.* (2018) to generate and characterize phenotypically SP- and SPHA-responsive T cells. TCC generated from serial dilution experiments were CD4+ and SP- and SPHA-proliferative responses were found to be HLA class II restricted. SP and SPHA activation of the TCC resulted in the release of Th1 and Th2 cytokines alongside the cytolytic molecules granzyme B and perforin. Like other sulfonamide metabolites, SPHA can spontaneously oxidize to a nitroso derivative (Coleman *et al.*, 1989; Rieder *et al.*, 1988; Uetrecht *et al.*, 1988; Winter and Unadkat, 2005). In fact, SPHA appears to be the least stable of parallel metabolites, and is therefore likely to be just as protein reactive (Pirmohamed *et al.*, 1991). Confirmation of thiol reactivity for the hydroxylamine/nitroso metabolite and detection of analogous adducts (Figure  3) to those seen with sulfamethoxazole and dapsone hydroxylamine/nitroso metabolites shows that these metabolites of SP can covalently bind biological macromolecules in a similar fashion (Alzahrani *et al.*, 2017; Naisbitt *et al.*, 1996), thereby acting as a hapten to elicit T-cell responses. However, as with the aforementioned sulfonamides, there exists controversy as to the identity of the critical drug entity responsible for SP hypersensitivity, with competing hapten and Pi hypotheses pertaining to T-cell responses arising to the parent drug and metabolites, respectively. Evidence for TCC proceeding through both of these pathways is provided in Figure  3. It has previously been speculated that these T-cell responses originate from the immunogenicity of the reactive hydroxylamine and nitroso metabolites based on findings from in vivo mouse studies (Alzahrani *et al.*, 2017), though as seen with sulfamethoxazole-responsive TCC, some T cells may well be specific for the parent sulfonamide (Castrejon *et al.*, 2010a). The cross-reactivity of SP-responsive TCC with SPHA appears to lend support to this, though this appears to be through a Pi interaction, and is therefore probably attributable to antigenic promiscuity, or alternatively, the reduction of the hydroxylamine metabolite back to SP within cultures.

**Figure 5.**
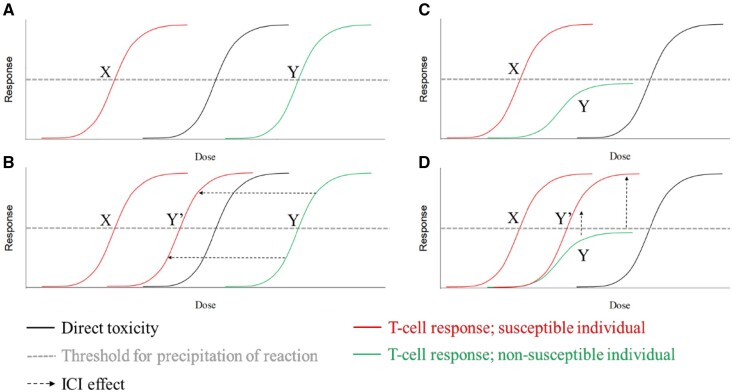
Dose dependence model for drug hypersensitivity reactions in susceptible and nonsusceptible individuals, and how ICIs impact upon the likelihood of an individual experience such a reaction. X, intrinsically susceptible individual; Y, nonsusceptible individual, Y′, individual in which ICIs confer hypersensitivity. A, B, Avidity model; ICIs confer a leftward shift upon the dose-response curve. C, D, Plateau model; regulatory mechanisms prevent T-cell responses from surpassing the threshold required for precipitation of hypersensitivity, ICIs alleviate this, resulting in an upward shift in the dose-response curve. A combination of both aspects may be applicable.

Two theoretical models to describe the increased susceptibility to drug hypersensitivity by ICIs within an otherwise tolerant individual are outlined in Figure  5. Figure  5A illustrates the dose dependency of aberrant T-cell responses to a compound, with the required antigen density for elicitation usually exceeding that of direct toxicity in the majority of individuals (governance determined by avidity-related factors such as antigen density, and immunological synapse components). The introduction of ICIs may result in T cells of lower avidity (and thus a larger overall compartment) being activated, resulting in a leftward shift to within a susceptible range (Figure  5B). In the second model (Figure  5C), the principle distinction is that T cells of susceptible individuals are able to mount responses of adequate magnitude to elicit hypersensitivity reactions, and that tolerance mechanisms prevent this from occurring in the majority of patients (as denoted by the plateau of response below the hypersensitive threshold). The net effect of the ICIs is to alleviate negative regulation of these responses, resulting in a hypersensitivity reaction as a product of otherwise tethered lymphocyte responses being allowed to manifest (Figure  5D). This appears to be supported by a clinical case study outlining the apparent checkpoint-induced disturbance of tolerance to contrast media and subsequent proof of concept studies on a memory antigen panel (Hammond *et al.*, 2021). It may be that a combination of both models applies, and that checkpoint blockade reduces both the avidity threshold and regulatory resistance resulting in propagation of aberrant responses to compounds. Regardless, it is apparent that the collateral effects of ICIs, through deregulation imparted through their mechanism of action, are effectuating the rise in immunological drug-drug interactions seen with increasing prevalence within the IO arena. Thus, as the field of IO comes of age in terms of implementation, so too must the management of patients in terms of concomitant medications.

## Notes

The authors certify that all research involving human subjects was done under full compliance with all government policies and the Helsinki Declaration.

## References

[kfab144-B1] Alzahrani A. , Ogese M., Meng X., Waddington J. C., Tailor A., Farrell J., Maggs J. L., Betts C., Park B. K., Naisbitt D. (2017). Dapsone and nitroso dapsone activation of naive T-cells from healthy donors. Chem. Res. Toxicol. 30, 2174–2186. 29045131 10.1021/acs.chemrestox.7b00263

[kfab144-B2] Aquino R. T. , Vergueiro C. S. V., Magliari M. E. R., Freitas T. H. (2008). Sulfasalazine-induced DRESS syndrome (drug rash with eosinophilia and systemic symptoms). Sao Paulo Med. J. 126, 225–226. 18853032 10.1590/S1516-31802008000400006 PMC11025977

[kfab144-B3] Castrejon J. L. , Berry N., El-Ghaiesh S., Gerber B., Pichler W. J., Park B. K., Naisbitt D. J. (2010a). Stimulation of human T cells with sulfonamides and sulfonamide metabolites. J. Allergy Clin. Immunol. 125, 411–418.e4. 20159253 10.1016/j.jaci.2009.10.031

[kfab144-B4] Castrejon J. L. , Lavergne S. N., El-Sheikh A., Farrell J., Maggs J. L., Sabbani S., O’Neill P. M., Park B. K., Naisbitt D. J. (2010b). Metabolic and chemical origins of cross-reactive immunological reactions to arylamine benzenesulfonamides: T-cell responses to hydroxylamine and nitroso derivatives. Chem. Res. Toxicol. 23, 184–192. 19954178 10.1021/tx900329b

[kfab144-B5] Chan M. M. , Kefford R. F., Carlino M., Clements A., Manolios N. (2015). Arthritis and tenosynovitis associated with the anti-PD1 antibody pembrolizumab in metastatic melanoma. J. Immunother. 38, 37–39. 25415286 10.1097/CJI.0000000000000060

[kfab144-B6] Chaput N. , Lepage P., Coutzac C., Soularue E., Le Roux K., Monot C., Boselli L., Routier E., Cassard L., Collins M., et al (2017). Baseline gut microbiota predicts clinical response and colitis in metastatic melanoma patients treated with ipilimumab. Ann. Oncol. 28, 1368–1379. 28368458 10.1093/annonc/mdx108

[kfab144-B7] Coleman M. , Breckenridge A., Park B. (1989). Bioactivation of dapsone to a cytotoxic metabolite by human hepatic microsomal enzymes. Br. J. Clin. Pharmacol. 28, 389–395. 2590600 10.1111/j.1365-2125.1989.tb03517.x PMC1379987

[kfab144-B8] Cui W. , Cotter C., Sreter K. B., Heelan K., Creamer D., Basu TN, Handy J., Walsh S., Popat S. (2020) Case of fatal immune-related skin toxicity from sequential use of osimertinib after pembrolizumab: Lessons for drug sequencing in never-smoking non-small-cell lung cancer. JCO Oncol. Pract. 16, 842–844. 32915710 10.1200/OP.20.00489

[kfab144-B9] Davidson A. , Bullowa J. G. (1940). Acquired hypersensitivity to sulfapyridine and sulfamethylthiazole. N. Engl. J. Med. 223, 811–813.

[kfab144-B10] Dubin K. , Callahan M. K., Ren B., Khanin R., Viale A., Ling L., No D., Gobourne A., Littmann E., Huttenhower C., et al (2016). Intestinal microbiome analyses identify melanoma patients at risk for checkpoint-blockade-induced colitis. Nat. Commun. 7, 10391. 26837003 10.1038/ncomms10391 PMC4740747

[kfab144-B11] Ford M. , Sahbudin I., Filer A., Steven N., Fisher B. A. (2018). High proportion of drug hypersensitivity reactions to sulfasalazine following its use in anti-PD-1-associated inflammatory arthritis. Rheumatology 57, 2244–2246. 30107548 10.1093/rheumatology/key234

[kfab144-B12] Gandhi S. , Pandey M., Ammannagari N., Wang C., Bucsek M. J., Hamad L., Repasky E., Ernstoff M. S. (2020). Impact of concomitant medication use and immune-related adverse events on response to immune checkpoint inhibitors. Immunotherapy 12, 141–149. 32064978 10.2217/imt-2019-0064 PMC7202260

[kfab144-B13] Giaffer M. , O'brien C., Holdsworth C. (1992). Clinical tolerance to three 5‐aminosalicylic acid releasing preparations in patients with inflammatory bowel disease intolerant or allergic to sulphasalazine. Aliment. Pharmacol. Ther. 6, 51–59. 1347468 10.1111/j.1365-2036.1992.tb00544.x

[kfab144-B14] Gibson A. , Faulkner L., Lichtenfels M., Ogese M., Al-Attar Z., Alfirevic A., Esser P. R., Martin S. F., Pirmohamed M., Park B. K., et al (2017a). The effect of inhibitory signals on the priming of drug hapten–specific T cells that express distinct Vβ receptors. J. Immunol. 199, 1223–1237. 28687658 10.4049/jimmunol.1602029 PMC5551967

[kfab144-B15] Gibson A. , Faulkner L., Wood S., Park B. K., Naisbitt D. J. (2017b). Identification of drug- and drug-metabolite immune responses originating from both naive and memory T cells. J. Allergy Clin. Immunol. 140, 578–581.e5. 28087328 10.1016/j.jaci.2016.11.032

[kfab144-B16] Gori S. , Inno A., Belluomini L., Bocus P., Bisoffi Z., Russo A., Arcaro G. (2019). Gut microbiota and cancer: How gut microbiota modulates activity, efficacy and toxicity of antitumoral therapy. Crit. Rev. Oncol. Hematol. 143, 139–147. 31634731 10.1016/j.critrevonc.2019.09.003

[kfab144-B17] Hammond S. , Gibson A., Jaruthamsophon K., Roth S., Mosedale M., Naisbitt D. J. (2020). Shedding light on drug-induced liver injury: Activation of T-cells from drug naive human donors with tolvaptan and a hydroxybutyric acid metabolite. Toxicol. Sci. 179, 95–107. 10.1093/toxsci/kfaa157 33078835

[kfab144-B18] Hammond S. , Olsson-Brown A., Gardner J., Thomson P., Ali S. E., Jolly C., Carr D., Ressel L., Pirmohamed M., Naisbitt D. (2021). T cell mediated hypersensitivity to previously tolerated iodinated contrast media precipitated by introduction of atezolizumab. J. Immunother. Cancer 9, e002521. 34049931 10.1136/jitc-2021-002521 PMC8166637

[kfab144-B19] Hargadon K. M. , Johnson C. E., Williams C. J. (2018). Immune checkpoint blockade therapy for cancer: An overview of FDA-approved immune checkpoint inhibitors. Int. Immunopharmacol. 62, 29–39. 29990692 10.1016/j.intimp.2018.06.001

[kfab144-B20] Hautekeete M. L. , Bourgeois N., Potvin P., Duville L., Reynaert H., Devis G., Adler M., Klöppel G. (1992). Hypersensitivity with hepatotoxicity to mesalazine after hypersensitivity to sulfasalazine. Gastroenterology 103, 1925–1927. 1360436 10.1016/0016-5085(92)91453-b

[kfab144-B21] Kimura H. , Hasegawa A., Takei I., Kawai T., Tsuchida Y., Abe Y., Hayashi R., Hama N., Abe R. (2020). Characteristic pathological features of keratinocyte death in a case of Stevens–Johnson syndrome manifested by an immune checkpoint inhibitor. J. Eur. Acad. Dermatol. Venereol. 35, e142–e145. 32780890 10.1111/jdv.16872

[kfab144-B22] Kittai A. S. , Oldham H., Cetnar J., Taylor M. (2017). Immune checkpoint inhibitors in organ transplant patients. J. Immunother. 40, 277–281. 28719552 10.1097/CJI.0000000000000180

[kfab144-B23] Koda R. , Watanabe H., Tsuchida M., Iino N., Suzuki K., Hasegawa G., Imai N., Narita I. (2018). Immune checkpoint inhibitor (nivolumab)-associated kidney injury and the importance of recognizing concomitant medications known to cause acute tubulointerstitial nephritis: A case report. BMC Nephrol. 19, 48. 29486725 10.1186/s12882-018-0848-y PMC5830324

[kfab144-B24] Leroux J. L. , Ghezail M., Chertok P., Blotman F. (1992). Hypersensitivity reaction to sulfasalazine: Skin rash, fever, hepatitis and activated lymphocytes. Clin. Exp. Rheumatol. 10, 427. 1356682

[kfab144-B25] Lomax A. J. , Lim J., Cheng R., Sweeting A., Lowe P., McGill N., Shackel N., Chua E. L., McNeil C. (2018). Immune toxicity with checkpoint inhibition for metastatic melanoma: Case series and clinical management. J. Skin Cancer 2018, 9602540. 29610684 10.1155/2018/9602540 PMC5828308

[kfab144-B26] Losek J. D. , Werlin S. L. (1981). Sulfasalazine hepatotoxicity. Am. J. Dis. Child. 135, 1070–1072. 6117199 10.1001/archpedi.1981.02130350070025

[kfab144-B27] Mak A. , Uetrecht J. (2015). The role of CD8 T cells in amodiaquine-induced liver injury in PD1–/–mice cotreated with anti-CTLA-4. Chem. Res. Toxicol. 28, 1567–1573. 26154582 10.1021/acs.chemrestox.5b00137

[kfab144-B28] McGahey K. E. , Weiss G. J. (2017). Reviewing concomitant medications for participants in oncology clinical trials. Am. J. Health Syst. Pharm. 74, 580–586. 28389457 10.2146/ajhp151052

[kfab144-B29] Metushi I. G. , Hayes M. A., Uetrecht J. (2015). Treatment of PD-1(-/-) mice with amodiaquine and anti-CTLA4 leads to liver injury similar to idiosyncratic liver injury in patients. Hepatology 61, 1332–1342. 25283142 10.1002/hep.27549

[kfab144-B30] Michot J. , Bigenwald C., Champiat S., Collins M., Carbonnel F., Postel-Vinay S., Berdelou A., Varga A., Bahleda R., Hollebecque A., et al (2016). Immune-related adverse events with immune checkpoint blockade: A comprehensive review. Eur. J. Cancer 54, 139–148. 26765102 10.1016/j.ejca.2015.11.016

[kfab144-B31] Naidoo J. , Cappelli L. C., Forde P. M., Marrone K. A., Lipson E. J., Hammers H. J., Sharfman W. H., Le D. T., Baer A. N., Shah A. A., et al (2017). Inflammatory arthritis: A newly recognized adverse event of immune checkpoint blockade. Oncologist 22, 627–630. 28576858 10.1634/theoncologist.2016-0390 PMC5469592

[kfab144-B32] Naisbitt D. J. , O'Neill P. M., Pirmohamed M., Park B. K. (1996). Synthesis and reactions of nitroso sulphamethoxazole with biological nucleophiles: Implications for immune mediated toxicity. Bioorg. Med. Chem. Lett. 6, 1511–1516.

[kfab144-B33] Ogese M. O. , Watkinson J., Lister A., Faulkner L., Gibson A., Hillegas A., Sakatis M. Z., Park B. K., Naisbitt D. J. (2020). Development of an improved T-cell assay to assess the intrinsic immunogenicity of haptenic compounds. Toxicol. Sci. 175, 266–278. 32159798 10.1093/toxsci/kfaa034

[kfab144-B34] Pezo R. C. , Wong M., Martin A. (2019). Impact of the gut microbiota on immune checkpoint inhibitor-associated toxicities. Ther. Adv. Gastroenterol. 12, 1756284819870911. 10.1177/1756284819870911 PMC6747860 31555343

[kfab144-B35] Pichler W. J. , Tilch J. (2004). The lymphocyte transformation test in the diagnosis of drug hypersensitivity. Allergy 59, 809–820. 15230812 10.1111/j.1398-9995.2004.00547.x

[kfab144-B36] Pirmohamed M. , Coleman M., Hussain F., Breckenridge A., Park B. (1991). Direct and metabolism‐dependent toxicity of sulphasalazine and its principal metabolites towards human erythrocytes and leucocytes. Br. J. Clin. Pharmacol. 32, 303–310. 1685664 10.1111/j.1365-2125.1991.tb03903.x PMC1368522

[kfab144-B37] Raithatha N. , Mehrtens S., Mouyis M., Manson J. (2014). Rash and fever after sulfasalazine use. BMJ 349, g5655. 25296685 10.1136/bmj.g5655

[kfab144-B38] Ransford R. , Langman M. (2002). Sulphasalazine and mesalazine: Serious adverse reactions re-evaluated on the basis of suspected adverse reaction reports to the Committee on Safety of Medicines. Gut 51, 536–539. 12235076 10.1136/gut.51.4.536 PMC1773410

[kfab144-B39] Rao S. , Cann P., Holdsworth C. (1987). Clinical experience of the tolerance of mesalazine and olsalazine in patients intolerant of sulphasalazine. Scand. J. Gastroenterol. 22, 332–336. 2884724 10.3109/00365528709078600

[kfab144-B40] Ribe J. , Benkov K. J., Thung S. N., Shen S. C., LeLeiko N. (1986). Fatal massive hepatic necrosis: A probable hypersensitivity reaction to sulfasalazine. Am. J. Gastroenterol. 81, 205–208. 2869683

[kfab144-B41] Rieder M. , Uetrecht J., Shear N., Spielberg S. (1988). Synthesis and in vitro toxicity of hydroxylamine metabolites of sulfonamides. J. Pharmacol. Exp. Ther. 244, 724–728. 3346843

[kfab144-B42] Shirali A. C. , Perazella M. A., Gettinger S. (2016). Association of acute interstitial nephritis with programmed cell death 1 inhibitor therapy in lung cancer patients. Am. J. Kidney Dis. 68, 287–291. 27113507 10.1053/j.ajkd.2016.02.057

[kfab144-B43] Sotolongo R. P. , Neefe L. I., Rudzki C., Ishak K. G. (1978). Hypersensitivity reaction to sulfasalazine with severe hepatotoxicity. Gastroenterology 75, 95–99. 45581

[kfab144-B44] Sullivan A. , Wang E., Farrell J., Whitaker P., Faulkner L., Peckham D., Park B. K., Naisbitt D. J. (2018). beta-Lactam hypersensitivity involves expansion of circulating and skin-resident TH22 cells. J. Allergy Clin. Immunol. 141, 235–249.e238. 28219704 10.1016/j.jaci.2017.01.020

[kfab144-B45] Turunen U. , Elomaa I., Anttila V.-J., Seppälä K. (1987). Mesalazine tolerance in patients with inflammatory bowel disease and previous intolerance or allergy to sulphasalazine or sulphonamides. Scand. J. Gastroenterol. 22, 798–802. 2890198 10.3109/00365528708991917

[kfab144-B46] Uetrecht J. , Zahid N., Shear N. H., Biggar W. D. (1988). Metabolism of dapsone to a hydroxylamine by human neutrophils and mononuclear cells. J. Pharmacol. Exp. Ther. 245, 274–279. 3129552

[kfab144-B47] Uhara H. , Kiyohara Y., Tsuda A., Takata M., Yamazaki N. (2018). Characteristics of adverse drug reactions in a vemurafenib early post-marketing phase vigilance study in Japan. Clin. Transl. Oncol. 20, 169–175. 28674996 10.1007/s12094-017-1706-2 PMC5797186

[kfab144-B48] Winter H. R. , Unadkat J. D. (2005). Identification of cytochrome P450 and arylamine N-acetyltransferase isoforms involved in sulfadiazine metabolism. Drug Metab. Dispos. 33, 969–976. 15843491 10.1124/dmd.104.002998

[kfab144-B49] Yamazaki N. , Uhara H., Fukushima S., Uchi H., Shibagaki N., Kiyohara Y., Tsutsumida A., Namikawa K., Okuyama R., Otsuka Y., et al (2015). Phase II study of the immune-checkpoint inhibitor ipilimumab plus dacarbazine in Japanese patients with previously untreated, unresectable or metastatic melanoma. Cancer Chemother. Pharmacol. 76, 969–975. 26407818 10.1007/s00280-015-2870-0 PMC4612320

[kfab144-B50] Zawodniak A. , Lochmatter P., Beeler A., Pichler W. J. (2010). Cross-reactivity in drug hypersensitivity reactions to sulfasalazine and sulfamethoxazole. Int. Arch. Allergy Immunol. 153, 152–156. 20413982 10.1159/000312632

